# Support-Free 3D Printing Based on Model Decomposition

**DOI:** 10.3390/mi16121316

**Published:** 2025-11-24

**Authors:** Xingguo Han, Qijin Qin, Shizheng Chen, Xuan Liu, Lixiu Cui

**Affiliations:** 1 School of Mechanical Engineering, Guangxi University, Nanning 530004, China; hanxingguo2004@163.com (X.H.); 2311391111@st.gxu.edu.cn (Q.Q.); 18120805329@163.cm (S.C.); 2 Guangxi Key Laboratory of Special Engineering Equipment and Control, Guilin 541004, China; 17863642183@163.com; 3 University Engineering Research Center of Non-Standard Intelligent Equipment and Process Control Technology, Guilin 541004, China; 4 Guangxi Region Precious Metal Materials Advanced Process Research Center, Guilin 541004, China; 5 School of Foreign Language and International Bussiness, Guilin University of Aerospace Technology, Guilin 541004, China

**Keywords:** additive manufacturing, volume decomposition, 3D printing, support-free

## Abstract

In traditional fixed-direction 3D printing, complex geometric models require support structures for large overhanging regions, leading to difficulties in removal, surface damage, and material waste. This study proposes a model decomposition algorithm for multi-axis 3D printing, aiming to divide the model into sub-regions requiring no or minimal support, which are printed sequentially via intermittent rotation of the print platform using a multi-DOF 3D printing device. Our algorithm employs a heuristic optimization method to minimize overhanging area and decomposition steps, leveraging Pareto multi-objective optimization to compute the Pareto front and adopting a beam search strategy to generate decomposition sequences. Decomposition and printing experiments on complex models demonstrate the method’s effectiveness in significantly reducing computational time, support requirements, and material costs while improving printing efficiency, enabling effective support-free multi-axis 3D printing.

## 1. Introduction

Additive manufacturing (AM) constructs complex geometric parts by depositing material layer by layer [1]. Due to its advantages in rapid prototyping, fabrication of complex geometries, and personalized production, AM has become a key technology in advanced manufacturing and is widely used in the automotive, aerospace, and biomedical fields [2,3,4].

**Figure 1:**
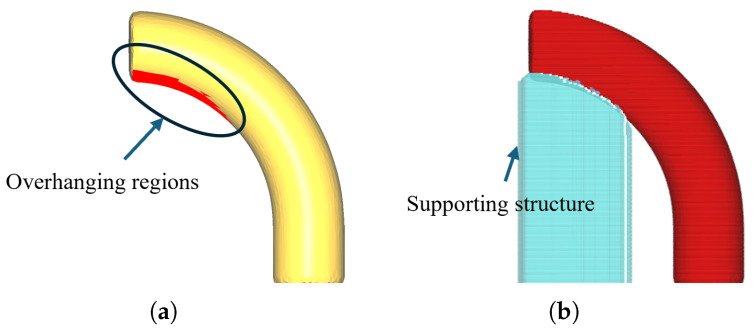
Overhang issues in 3-axis printing. (**a**) Bend pipe model with overhanging region. (**b**) The overhanging area requires the addition of support structures.

Currently, most industrial AM systems are still based on traditional three-axis manufacturing technology. These devices are typically limited to motion along the X, Y, and Z axes, constraining material deposition to a single, fixed direction (usually the +Z direction). This fundamental limitation poses a core challenge: support structures must be introduced when manufacturing parts with overhangs (as shown in Figure 1) to prevent the collapse of material during deposition [5]. The use of support structures not only increases material consumption and printing time but also introduces a cumbersome post-processing task for their removal, which raises manufacturing costs and can potentially damage the final surface quality of the printed part [6].

In recent years, to fundamentally address the issues caused by support structures, multi-axis printing has emerged as a critical solution. By dynamically adjusting the deposition direction through additional degrees of freedom, multi-axis systems enable each region of a complex geometry to be printed in an optimal, support-free orientation. Correspondingly, various multi-degree-of-freedom systems have been developed, including a five-axis CNC printing system by Pan et al. [7], a hybrid robotic platform by Keating and Oxman [8], a Stewart parallel mechanism by Song et al. [9], and other multi-DOF systems [10,11,12].

Leveraging the flexibility of multi-axis systems for support-free printing presents a core computational challenge at the software level: how to optimally decompose a 3D digital model and plan paths for multi-directional printing. This process is commonly referred to as volumetric decomposition, and current research primarily evolves along two technical pathways:

Layer-based decomposition aims to generate globally continuous non-planar printing layers. By constructing curved layers that conform to the model geometry, these methods achieve support-free fabrication. Representative methods include geodesic-based curved layering [12,13], ellipsoidal decomposition [14], thermal-field isothermal layering [15], and rotation-driven scalar-field layering [16]. They generally provide excellent surface quality and interlayer bonding strength. However, these methods share a common bottleneck: the continuously and slowly varying build direction drastically reduces deposition efficiency and substantially increases motion-control complexity. Moreover, with the exception of ellipsoidal decomposition [14], all other curved-layer methods produce concave regions that are highly susceptible to local nozzle gouging.

Part-based decomposition, in contrast, divides the model into several sub-modules, each of which uses fixed-direction planar layered printing. Intermittently adjusting the deposition direction reduces support and improves efficiency. Early research, such as the Chopper framework proposed by Luo et al. [17], primarily aimed to decompose large parts to fit within limited printing volumes, though it required post-printing assembly. Subsequent research focused on achieving support-free fabrication, exemplified by Wei et al.’s skeleton-based algorithm [18] and Hu et al.’s near-pyramidal decomposition [19]. Biet et al. [20] further expanded the optimization objectives to simultaneously achieve support-free printing and enhanced structural strength. The series of work by Wu et al. marks a significant advancement in this domain: they formalized the search for an optimal sequence of cutting planes as a computational search problem, introducing a beam-guided search algorithm [21]. Although subsequent work focused on accelerating this search process [22], current planar decomposition research struggles to accommodate more diverse geometric model structures and often suffers from low computational efficiency, as seen in methods like [5,21].

This study proposes an efficient model decomposition algorithm that aims to decompose a model into subcomponents with no or minimal supports by generating a sequence of cutting planes. An AM device based on a six-DOF robotic arm was built to ensure that the material deposition direction is perpendicular to the part printing layer, enabling support-free manufacturing of parts, thereby reducing material costs and improving manufacturing efficiency.

## 2. Methods

### 2.1. Problem Modeling

**Figure 2:**
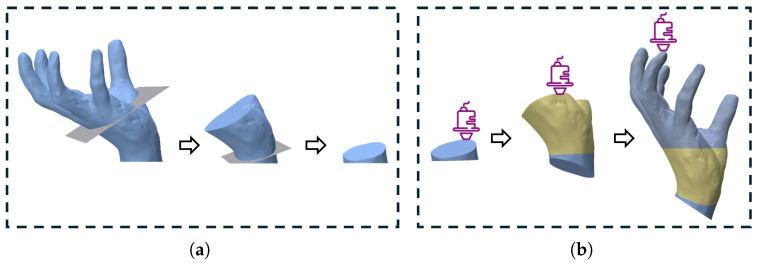
Example of our algorithm implementing support-free printing. (**a**) Disassembling the model piece by piece according to the cutting plane sequence numbers. (**b**) Printing the parts in reverse order of disassembly.

Given a solid model *M* with printing orientation d, using a set of ordered sequences of cutting planes $\left\{p_{i},i=0,\ldots ,n\right\}$, sequentially cutting and decomposing *M* step by step can decompose the solid model *M* into n+2 components $\left\{M_{i},i=0,\ldots ,n+1\right\}$, as shown in Figure 2a.

After decomposing the solid model *M* into individual components requiring no support structures, each component is printed sequentially in reverse order of cutting. The print direction of each component $M_{i}$ (i=0,…,n) is aligned with the normal vector of its corresponding cutting plane $p_{i}$ (where $M_{n+1}$ is printed along direction d). During printing, after each component is completed, the printing platform rotates to build the next block upon the foundation of the preceding one. This ultimately enables support-free printing of the entire model *M*, as shown in Figure 2b.

Therefore, to generate a support-free decomposition that satisfies *M*, we need to find a sequence of cutting planes such that each component cut by each plane has no overhang area or as little overhang area as possible in its corresponding print orientation.

The self-supporting state of *f* on component $M_{i}$ is determined by the angle between its normal vector $n_{f}$ and the corresponding printing direction $V_{i}$, which is defined as

(1) $$e\left(f,V_{i}\right)=\left\{\begin{array}{cc} 1, & V_{i}\cdot n_{f}+\sin\alpha_{\max}<0\\ 0, & \mathrm{otherwise} \end{array}\right.$$

where $\alpha_{\max}$ represents the maximum self-supporting angle (ref. [6]).

The overhang area of each component $M_{i}$ relative to its printing orientation can be expressed as follows:

(2) $$S_{overhang,i}=\sum_{f\in M_{i}}e\left(f,V_{i}\right)S\left(f\right)$$

where S(f) represents the area of face *f*. To minimize the overhang area during model printing, the objective function is defined as follows:

(3) $$S=\min\sum_{i=0}^{n+1}S_{overhang,i}$$

Typically, the surface patches of model parts are sufficiently small, and their areas differ only slightly. To facilitate computational efficiency, we transform the calculation of overhang area into determining the number of critical surface patches, converting the minimization of overhang area into minimizing the number of critical overhang patches.

The model serves as input for algorithmic solutions. We extract the non-bottom faces of the model—those not in contact with the print platform—since the bottom faces need not participate in this computational process. These faces are represented in matrix form, where each face’s position is defined by the centers of its three vertices. This yields the position matrix A, while the face normals are represented by the normal matrix B. Both A and B are *N*-dimensional 3-column matrices. Each row corresponds to the position and normal of a single face, where *N* is the total number of faces.

The cutting plane can be defined by its normal vector v(a,b,c) and scale factor *l*, as shown in Equation (4).

(4) $$\left(a,b,c\right)\cdot {\left[x,y,z\right]}^{T}=l$$

This equation represents the plane normal vector as a column vector v with three rows. Then, if $A_{j}\cdot v-l>0$, then surface patch *j* lies above the plane (we define the plane’s normal direction as pointing upward; the opposite direction points downward). If $B_{j}\cdot v+\sin\alpha_{\max}>0$, then the angle between surface patch *j* and the plane’s normal satisfies the support requirement. For all surface patches above the plane to meet the support requirement, the following must hold:

(5) $$B_{j}\cdot v+\sin\alpha_{\max}>0\;\forall j\in \left\{f_{j}\;|\;A_{j}\cdot v-l>0\right\}$$

For convenience, in the cutting plane sequence, we denote each plane as $p_{i}\left(v_{i},l_{i}\right)$. We use the column vector $C(p_{i})$ to represent the positional relationship between the model’s facets and the plane $p_{i}$ during cutting:

(6) $$C\left(p_{i}\right)=H\left(A\cdot v_{i}-l_{i}\cdot I\right)$$

where I is an *N*-dimensional all-ones column vector, and H(x) is the Heaviside function. The column vector $D(p_{i})$ represents the angular relationship between the facet normals and the plane normal during cutting by $p_{i}$:

(7) $$D\left(p_{i}\right)=H\left(B\cdot v_{i}+\sin\alpha_{\max}\cdot I\right)$$

Since the model is represented by the facet matrices A and B, with each row corresponding to a facet’s position and normal, we use a binary mask vector mask to indicate the active state (0 or 1) of each facet. For the initial model, ${\mathrm{mask}}_{0}=I$. The state update formula for the model after the *i*-th cut in the plane sequence is

(8) $${\mathrm{mask}}_{i+1}={\mathrm{mask}}_{i}\circ \left(I-C\left(p_{i}\right)\right)$$

Thus, the objective of the cutting plane sequence can be formulated as

(9) $$S=\min_{{\left\{v_{i},l_{i}\right\}}_{i=0}^{n}}\left[\sum_{i=0}^{n}{\left({\mathrm{mask}}_{i}\circ C\left(p_{i}\right)\right)}^{T}\cdot \left(I-D\left(p_{i}\right)\right)+{\mathrm{mask}}_{n+1}\cdot \left(I-\Gamma \right)\right]$$

where ∘ denotes the Hadamard product, *n* is the total number of planes in the cutting sequence, and $\Gamma =H(B\cdot d+\sin\alpha_{\max}\cdot I)$.

Equation (9) quantifies the total number of facets still requiring support after model decomposition, with the optimization goal of minimizing the number of overhanging facets. However, Equation (9) is based on the initial model’s facets and does not account for updates to hole boundary facets after cutting. During optimization, new facets at hole boundaries may require support if the angle between cutting planes exceeds the maximum self-supporting angle $\alpha_{\max}$. We address this by imposing constraints on the cutting planes (see Section 2.2.1).

### 2.2. Optimization Strategy

The challenge in solving Equation (9) lies in the unknown number of planes *n* in the cutting plane sequence, with each plane defined by its normal v and offset *l*, and the need to consider plane ordering, leading to a complex search space that renders traditional global optimization inefficient. To address this, we employ an iterative optimization strategy. First, we generate a discrete set of candidate planes, use a Pareto multi-objective optimization algorithm to select the optimal plane set, construct sequences from the planes in the set, update the model state, and continue with the next round of selection until the remaining sub-regions are nearly support-free. Finally, we identify the optimal sequence from all generated sequences.

#### 2.2.1. Candidate Plane Generation and Constraints

**Figure 3:**
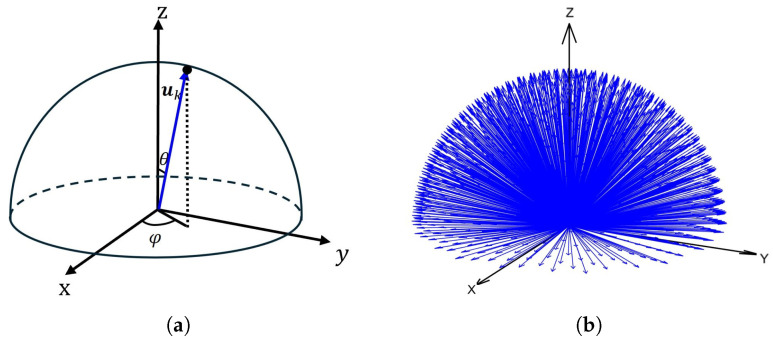
Generation of plane normal vectors: (**a**) definition of longitude $\phi_{k}$ and latitude $\theta_{k}$ on a hemisphere; (**b**) Fibonacci spherical sampling results.

The generation of candidate planes is critical to the solution quality, and we apply constraints to them. We use unit sphere sampling to generate plane normals, employing Fibonacci spherical sampling on the upper hemisphere (z-axis) of the unit sphere [23], determined by two variables $\theta_{k}$ and $\phi_{k}$, as shown in Figure 3a. The corresponding $u_{k}$ is computed according to Equation (10), yielding *K* plane normal vectors, with *K* set to 1000 in our algorithm, as shown in Figure 3b.

(10) $$u_{k}={\left(\sin\theta_{k}\cos\phi_{k},\;\sin\theta_{k}\sin\phi_{k},\;\cos\theta_{k}\right)}^{T}$$

where $\theta_{k}=\arccos\left(1-\frac{2k+1}{2K+1}\right),k=0,1,2,\ldots ,K$, $\phi_{k}=\frac{2\pi k}{\phi_{\mathrm{golden}}}$, $\phi_{\mathrm{golden}}=\frac{1+\sqrt{5}}{2}$.

**Figure 4:**
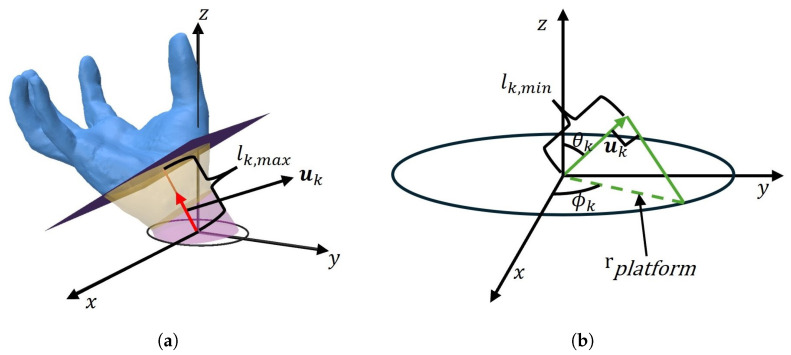
Subject to the constraint *l*: (**a**) all model surface patches on plane $q(u_{k},l_{k,max})$ are self-supporting; (**b**) plane $q(u_{k},l_{k,min})$ must not intersect the circular platform with radius $r_{\mathrm{platform}}$ at the model’s base.

For $u_{k}$, we compute $l_{k,\max}$ to define the plane $p(u_{k},l_{k,\max})$, ensuring that all facets above the plane are self-supporting and that the plane covers the maximum number of non-overhanging facets, as shown in Figure 4a. $l_{k,\max}$ is obtained via Equation (11):

(11) $$l_{k,\max}=\min_{j:B_{j}\cdot u_{k}+\sin\alpha_{\max}<0}\left(A_{j}\cdot u_{k}\right)$$

To prevent the print head from colliding with the platform, no cutting plane is allowed to intersect the model’s bottom, as shown in Figure 4b. We define a circular platform with radius $r_{\mathrm{platform}}$ at the model’s base, and for $u_{k}$, the minimum offset $l_{k,\min}$ is computed via Equation (12):

(12) $$l_{k,\min}=r_{\mathrm{platform}}\cdot \sin\theta_{k}$$

Thus, the offset *l* for each $u_{k}$ lies in the interval $\left\{l_{k,\min}<l_{k,m}\leq l_{k,\max}\right\}$. By generating offset values $\left\{l_{k,m}\right\}$ ($m=1,2,\ldots ,M_{k}$) with discrete steps, we form the candidate plane set $Q=\{q_{k,m}\left(u_{k},l_{k,m}\right)|k=0,\ldots ,K,m=1,\ldots ,M_{k}\}$.

Since A and B only account for the original model’s facets, when a plane $p_{i}$ cuts the model, the angle between $p_{i}$ and other planes in the sequence may exceed the maximum self-supporting angle, causing the component $M_{i}$ to require support. Specifically, when the cut sub-region $M_{i}$ includes facets filled by planes in the sequence, and the angle between two planes exceeds the maximum self-supporting angle, overhanging facets arise. To address the impact of hole boundary facets introduced by cutting, we impose plane constraints. The boundary facet vector for the cutting plane $p_{i}$ in the sequence is defined:

(13) $$\mathrm{boundary}\left(p_{i}\right)=H\left(A\cdot v_{i}-\left(l_{i}-\varepsilon \right)\cdot I\right)$$

where ε is a constant value of 1.

The overhanging condition between candidate plane $q_{k,m}$ and all planes $p_{i}$ in the current sequence is computed:

(14) $$\delta \left(q_{k,m},p_{i}\right)=u_{k}^{T}\cdot v_{i}+\sin\alpha_{\max}$$

If $\delta (q_{k,m},p_{i})<0$ exists, the boundary coverage of plane $q_{k,m}$ under the current model state should be computed using Equation (15). If $\zeta (q_{k,m},p_{i})>0$, plane $q_{k,m}$ should be removed.

(15) $$\zeta \left(q_{k,m},p_{i}\right)={\left({\mathrm{mask}}_{i}\circ C\left(q_{k,m}\right)\right)}^{T}\cdot \mathrm{boundary}\left(p_{i}\right)$$

#### 2.2.2. Evaluation Metrics and Pareto Optimization

To select the optimal plane set from the candidate plane set *Q*, we define three evaluation metrics:

Support Reduction Rate $S_{r}\left(p_{i}\right)$: For a model, overhanging facets requiring support arise due to angles with the deposition direction exceeding the maximum self-supporting angle. We define the proportion of overhanging facets removed by a plane relative to the total overhanging facets in the current model as the support reduction rate. A higher ratio indicates more overhanging facets removed, reducing the number of subsequent cuts needed.
  (16) $$S_{r}\left(p_{i}\right)=\frac{{\left({\mathrm{mask}}_{i}\circ C\left(p_{i}\right)\right)}^{T}\cdot \left(I-\Gamma \right)}{{\mathrm{mask}}_{i}^{T}\cdot \left(I-\Gamma \right)}$$
  Support Ratio $S_{i}\left(p_{i}\right)$: This is the proportion of facets above plane $p_{i}$ with angles exceeding the maximum self-supporting angle relative to the total facets. For models where zero support is unachievable, we introduce progressive relaxation, gradually loosening the support ratio constraint to ensure that the decomposition process continues.
  (17) $$S_{i}\left(p_{i}\right)=\frac{{\left({\mathrm{mask}}_{i}\circ C\left(p_{i}\right)\right)}^{T}\cdot \left(I-D\left(p_{i}\right)\right)}{{\left({\mathrm{mask}}_{i}\circ C\left(p_{i}\right)\right)}^{T}\cdot I}$$
  Facet Coverage Rate $R_{c}\left(p_{i}\right)$: This is the proportion of facets above plane $p_{i}$ relative to the total facets in the current model. A higher value indicates fewer remaining facets, reducing the need for subsequent cuts.
  (18) $$R_{c}\left(p_{i}\right)=\frac{{\left({\mathrm{mask}}_{i}\circ C\left(p_{i}\right)\right)}^{T}\cdot I}{{\mathrm{mask}}_{i}^{T}\cdot I}$$

Based on these metrics, we select the optimal planes from the candidate set *Q* using Pareto optimization [24], considering the support reduction rate $S_{r}\left(q_{k,m}\right)$ (maximized), support ratio $S_{i}\left(q_{k,m}\right)$ (minimized), and facet coverage rate $R_{c}\left(q_{k,m}\right)$ (maximized). First, we construct the objective vector:

(19) $$f\left(q_{k,m}\right)={\left(S_{r}\left(q_{k,m}\right),\;-S_{i}\left(q_{k,m}\right),\;R_{c}\left(q_{k,m}\right)\right)}^{T},\;q_{k,m}\in Q$$

The dominance relation between planes is defined. Plane $q_{a}$ dominates $q_{b}$ (denoted as $q_{a}≺q_{b}$) if and only if $q_{a}$ is better than $q_{b}$ in at least one metric and not worse in others, and their metric vectors differ:

(20) $$q_{a}≺q_{b}\Leftrightarrow \left\{\begin{array}{c} S_{r}\left(q_{a}\right)\geq S_{r}\left(q_{b}\right)\\ S_{i}\left(q_{a}\right)\leq S_{i}\left(q_{b}\right)\\ R_{c}\left(q_{a}\right)\geq R_{c}\left(q_{b}\right)\\ f\left(q_{a}\right)\neq f\left(q_{b}\right) \end{array}\right.$$

By comparing the objective vectors $f(q_{k,m})$ of each candidate plane $q_{k,m}$ in set *Q*, we select all non-dominated planes to form the set:

(21) $$P=\{q_{k,m}\in Q|∄q^{\prime }\in Q\;s.t.\;q^{\prime }≺q_{k,m}\}$$

The set P is the Pareto front, with planes in P achieving the optimal trade-off among the three metrics.

#### 2.2.3. Beam Search and Sequence Generation

To generate a sequence of cutting planes for support-free printing, we employ a beam search of width *b* [25]. The algorithm initializes *b* empty beams, each of which maintains a plane sequence $P=\{p_{i},i=0,\ldots ,n\}$ and a model state ${\mathrm{mask}}_{i}$. First, a set of candidate planes *Q* is generated. The Pareto front P is selected using Equations (19) and (21). The constraint check (δ, ζ) is then performed to remove invalid planes. If |P|>b, the first *b* planes with the minimum $S_{i}\left(q_{k,m}\right)$ are retained, ${\mathrm{mask}}_{i}$ is updated, and the *b* beams are filled. If the residual support $S_{\mathrm{res}}\left({\mathrm{mask}}_{i+1}\right)=\frac{{\mathrm{mask}}_{i+1}^{T}\cdot \left(I-\Gamma \right)}{{\mathrm{mask}}_{i+1}^{T}\cdot I}>0.001$, the corresponding beam is further screened for *b* planes, generating at most $b^{2}$ candidate sequences. These sequences are sorted by total support $\sum S_{i}\left(p_{i}\right)$, and the first *b* sequences are retained to populate the beam. Iterations continue until all beams satisfy Sres<0.001, the maximum number of iterations is reached, or no valid solution exists. From the *b* beams, the optimal sequence with the minimum $\sum S_{i}\left(p_{i}\right)$ and the fewest planes is selected. The complete workflow is shown in Algorithm 1.


**Algorithm 1:** Model Decomposition.

    **Input**: Mesh model    **Output**: Optimal plane sequence $P=\{p_{i}\}$ 1:Initialize *A*, *B*, Compute $r_{\mathrm{platform}}$ 2:
**====== Candidate Plane Generation ======**
 3:

Q←∅

 4:**for **k=0 to K−1 **do** 5:    $u_{k}\leftarrow {\left(\sin\theta_{k}\cos\phi_{k},\sin\theta_{k}\sin\phi_{k},\cos\theta_{k}\right)}^{T}$ 6:    $l_{k,\max}\leftarrow \min_{j:B_{j}\cdot u_{k}+\sin\alpha_{\max}<0}\left(A_{j}\cdot u_{k}\right)$ 7:    $l_{k,\min}\leftarrow r_{\mathrm{platform}}\cdot \sin\theta_{k}$ 8:    **for** m=1 to $M_{k}$ **do** 9:        $l_{k,m}\leftarrow l_{k,\min}+\left(m-1\right)\cdot \Delta l$10:        $Q\leftarrow Q\cup \{\left(u_{k},l_{k,m}\right)\}$11:    **end for**12:
**end for**
13:Initialize *b* empty beams: P=∅, ${\mathrm{mask}}_{0}$, support-free = false14:
**====== Beam Search Expansion ======**
15:**for **t=1 to *T* **do**16:    **for** each beam with support-free = false in current *b* beams **do**17:        **====== Plane Selection ======**18:        Compute $S_{r}\left(q\right)$, $S_{i}\left(q\right)$, $R_{c}\left(q\right)$ for all q∈Q19:        P←ParetoFront(Q)20:        **for** each q∈P **do**21:           **for** each $p_{i}\in P$ **do**22:               Compute $\delta (q,p_{i})$, $\zeta (q,p_{i})$23:               **if** δ<0 and ζ>0 **then**24:                   Remove *q* from P25:               **end if**26:           **end for**27:        **end for**28:        **if** |P|<b **then**29:           Generate |P| new beams30:        **else**31:           Sort by $S_{i}$, retain top *b* planes, generate *b* new beams32:        **end if**33:        Update ${\mathrm{mask}}_{i+1}$, $B_{\mathrm{new}}$34:    **end for**35:    **=== Termination Check ===**36:    **if** $|B_{\mathrm{new}}|=0$ or all beams support-free or t=T **then**37:        **break**38:    **end if**39:
**end for**
40:Select optimal sequence with minimal $\sum S_{i}\left(p_{i}\right)$41:
**return** *P*





## 3. Multi-Degree-of-Freedom 3D Printing

**Figure 5:**
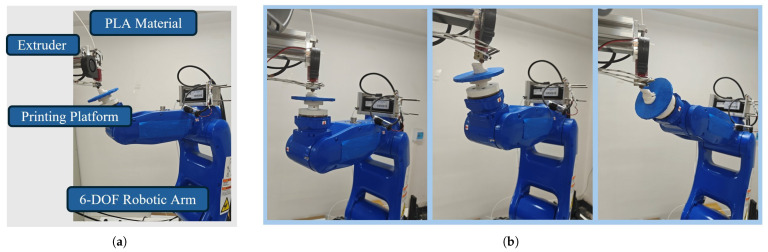
Printing system hardware and its model printing process. (**a**) Printing system hardware configuration; (**b**) Printing process of the model implemented on our robotic arm.

We utilize a six-degree-of-freedom (6-DOF) robotic arm (model GP8, with a payload of 8 kg and a repeatability accuracy of 20 µm) to construct a multi-axis fused deposition modeling (FDM) printing device. In our hardware setup, the print platform is mounted at the end of the GP8 robotic arm (Yaskawa Motoman, Kitakyushu, Japan), while the extruder is fixed to the frame, with the nozzle extrusion direction maintained along the gravity direction. We keep the extruder’s pose constant and achieve multi-directional material deposition by controlling the pose of the print platform at the robotic arm’s end, as shown in Figure 5.

**Figure 6:**
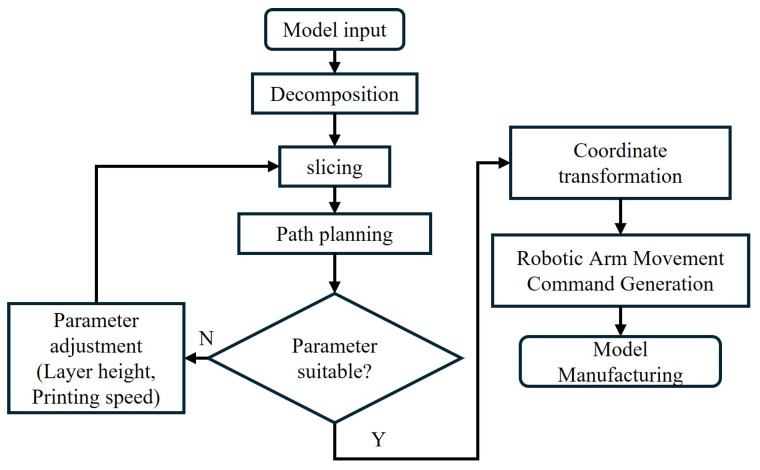
Steps for system manufacturing modeling.

The robotic arm’s movements throughout the entire printing process are executed by the robot motion-control program. Therefore, the model to be printed must be converted into a motion control file for the robotic arm. The primary workflow involves operations such as model decomposition, layer slicing, path planning, and coordinate transformation. Once the robotic arm’s motion file is generated, it is transmitted to the robotic arm’s control module to complete the model fabrication. The specific steps for the printing system to manufacture the model are illustrated in Figure 6.

**Figure 7:**
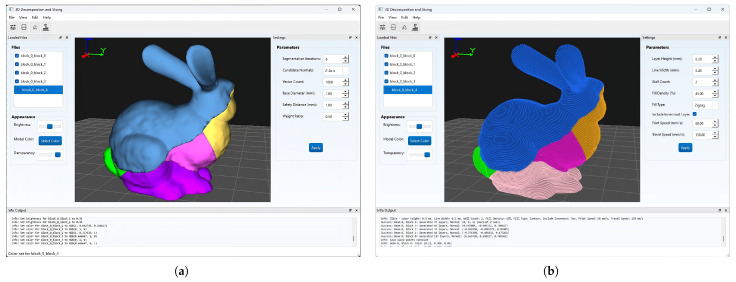
Three-dimensional decomposition and slicing software based on our decomposition algorithm: (**a**) model decomposition; (**b**) hierarchical organization and path planning based on decomposition results.

We implemented the algorithm and developed software capable of model decomposition and slicing. When a model is imported into our 3D decomposition and slicing software, the algorithm solves for a sequence of cutting planes to decompose the model. Upon completion, individual components are generated, as shown in Figure 7a. The software integrates slicing functionality, allowing users to configure slicing parameters. It then layers the decomposed components according to the normal direction of the corresponding cutting planes and completes path planning, as shown in Figure 7b.

### Coordinate Transformation

**Figure 8:**
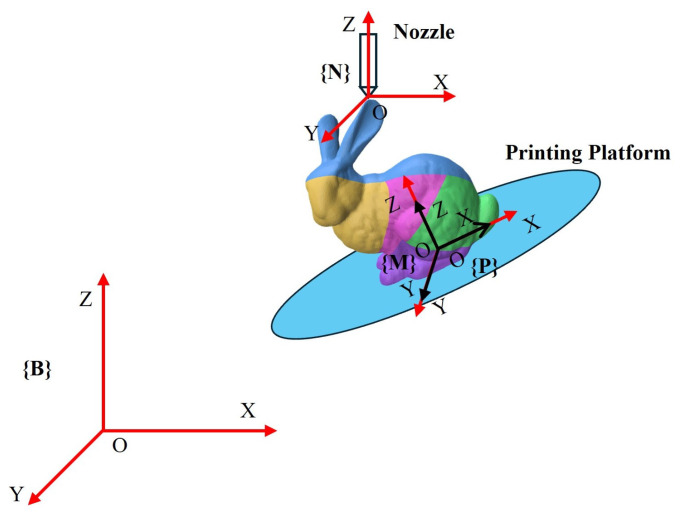
Coordinate system used in robot printing.

Since the slice path represents the tool path, we need to convert it into the pose path of the printing platform connected to the robotic arm. The core principle is simple: for each printing point, the platform rotates and translates so that the point is directly below the fixed nozzle, and its normal (the printing direction of the part containing that point) points upwards. This system contains four coordinate systems (Figure 8):

- Base Coordinate System {B}: Fixed on the robotic arm base.
- Model Coordinate System {M}: Defines the geometry of the 3D model.
- Platform Coordinate System {P}: Origin located at the center of the printing platform.
- Nozzle Coordinate System {N}: Fixed at the nozzle tip, with its Z-axis upward (${\left[0,0,1\right]}^{T}$).

For ease of calculation, the model coordinate system {M} is aligned with the platform coordinate system {P}. The transformation is performed in three steps:

1. Direction Alignment: The platform is rotated so that the normal $n_{i}$ of the current print point is aligned with the nozzle’s z-axis direction:
  (22) $$R_{B}^{P}\cdot n_{i}={\left[0,0,1\right]}^{T}$$
  $R_{B}^{P}$ can be obtained using the standard axis-angle (Rodrigues) formula.
2. Safety Adjustment: The system itself has redundant degrees of freedom. We added a rotation ϕ about the Z-axis to orient the platform from the robotic arm position toward the nozzle (see Figure 5b), while preventing the robotic arm from exceeding the joint movement limits and collisions between the link and the nozzle support:
  (23) $$R=R_{z}\left(\psi \right)\cdot R_{B}^{P}$$
  where $\psi =atan2\left(n_{ix}-\frac{p_{nx}}{p_{ny}}\cdot n_{iy},\;n_{iy}+\frac{p_{nx}}{p_{ny}}\cdot n_{ix}\right)$.
3. Position Calculation: The platform is translated so that the print point coincides with the nozzle tip:
  (24) $$t_{B}^{P}=p_{n}-R\cdot p_{i}$$
  This transformation ensures that the print platform deposits material correctly and achieves collision-free movement throughout the printing process.

## 4. Results and Discussion

**Figure 9:**
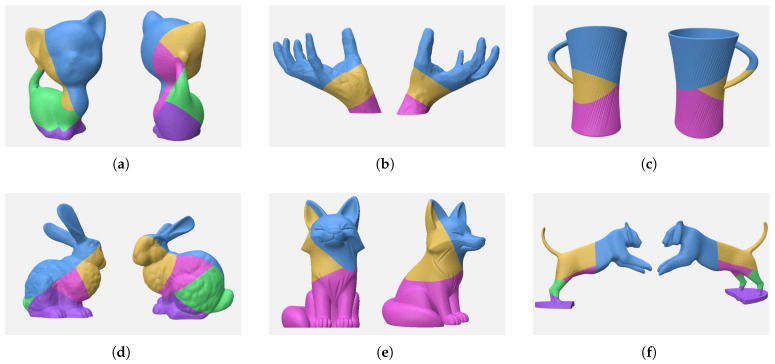
Decomposition results of our algorithm, where each sub-figure shows two views of the decomposed model: (**a**) Kitten; (**b**) Hand; (**c**) Cup; (**d**) Bunny; (**e**) Fox; (**f**) Tiger.

We implemented the proposed algorithm using C++17 and enabled OpenMP parallel computing on a PC equipped with an Intel 13400F CPU and 16 GB of RAM. The algorithm parameters were set as follows: beam width b=15, maximum iterations T=6, discrete step Δl=1 mm, number of direction vectors K=1000, and maximum self-supporting angle $\alpha_{\max}=45^{\circ }$. A 0.4 mm nozzle and PLA filament were used for all printing experiments. To demonstrate the effectiveness of our algorithm, we applied the decomposition algorithm to various models, with the results of the decomposition for different models shown in Figure 9.

**Figure 10:**
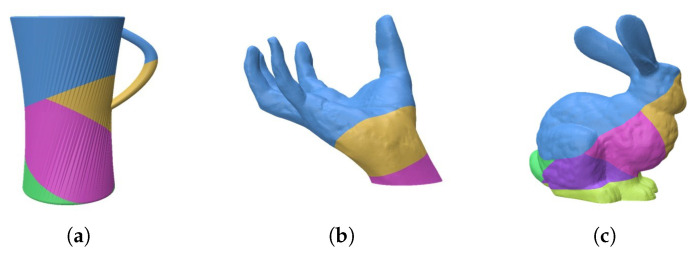
Wu’s accelerated decomposition algorithm results: (**a**) Cup; (**b**) Hand; (**c**) Bunny.

**Computation time.** Our algorithm significantly reduces the computational complexity of plane screening through matrix representation and vector calculations, and with OpenMP parallel optimization, it markedly improves decomposition efficiency. We ran our algorithm and Wu’s [22] accelerated decomposition algorithm on our PC equipped with an Intel 13400F CPU and 16 GB of RAM, solving models such as Bunny, Hand, and Cup. Our algorithm’s average computation time is only 6.4 s (with computation times for model solving reported in Table 1), representing an approximately 88% reduction compared to Wu’s accelerated decomposition algorithm, which has an average computation time of about 56 s (with specific times of 82.1 s for Bunny, 51.1 s for Hand, and 36.5 s for Cup). The decomposition results of Wu’s algorithm are shown in Figure 10. Both algorithms achieve unsupported decomposition for all three models, but our algorithm decomposes the Bunny and Cup with fewer components and has significantly less computational time.

**Overhanging area.** Our algorithm does not explicitly calculate the area of risk regions requiring support during the computation process; instead, it uses the number of supported facets as the optimization metric. After obtaining the plane sequence through the algorithm, we calculated the risk area, as shown in Table 1. Compared to the original models, the overhanging area of the decomposed models is significantly reduced. While some models cannot achieve zero overhanging area, the algorithm still generates decomposition results, reducing the overhanging area by more than 95%.

**Figure 11:**
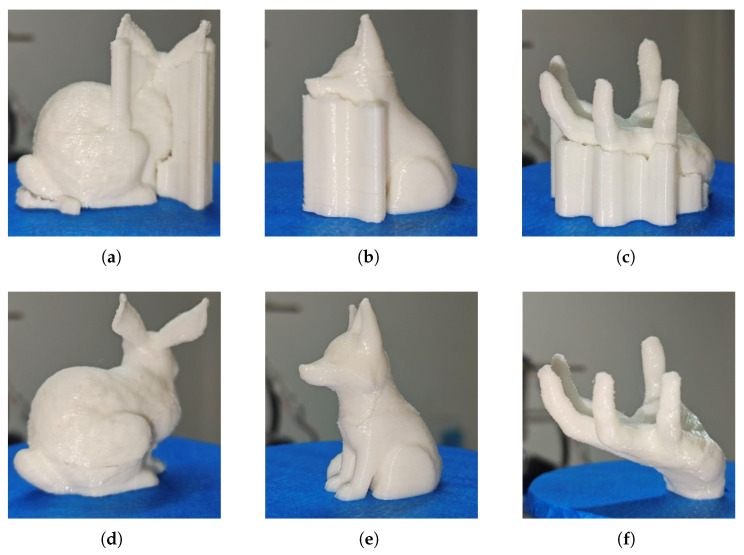
Comparison of fixed-direction printing and multi-directional printing: (**a**) Bunny, (**b**) Fox, and (**c**) Hand show fixed-direction printing, while (**d**) Bunny, (**e**) Fox, and (**f**) Hand show multi-directional printing after algorithmic decomposition.

**Material savings.** Our algorithm aims to achieve support-free printing whenever possible to reduce material costs. To evaluate its effectiveness, we compared material usage between fixed-direction printing (Fixed Dir) and multi-directional printing (Multi-Dir) after algorithmic decomposition, using identical slice thicknesses. We sliced the Bunny, Fox, and Hand models, with a layer height of 0.25 mm and 45% zigzag infill. We then conducted printing experiments on the three models, with the results shown in Figure 11. Table 2 summarizes material consumption (expressed as filament length) for the three models under fixed-direction and multi-directional printing. In our experiments, multi-directional printing using our method reduced material consumption by approximately 20% compared to fixed-direction printing with support structures. Specifically, the filament length of the Fox model was reduced by 22%, while the filament lengths of the Bunny and Hand models were reduced by 17.4% and 23.9%, respectively.

**Printing time.** Using plane decomposition followed by multi-directional printing, the platform rotates only during the transition between two components, with transition times being extremely short (in the order of seconds). Thus, the printing time is primarily influenced by the total length of the tool path. We conducted printing experiments on the Bunny, Fox, and Hand models, with results shown in Figure 11. Table 2 reports the printing times for the Bunny, Fox, and Hand models in fixed-direction printing and multi-directional printing after decomposition with our algorithm. In our experiments, under the same slice thickness, the printing time for multi-directional printing after decomposition with our algorithm was reduced by an average of approximately 20%.

**Figure 12:**
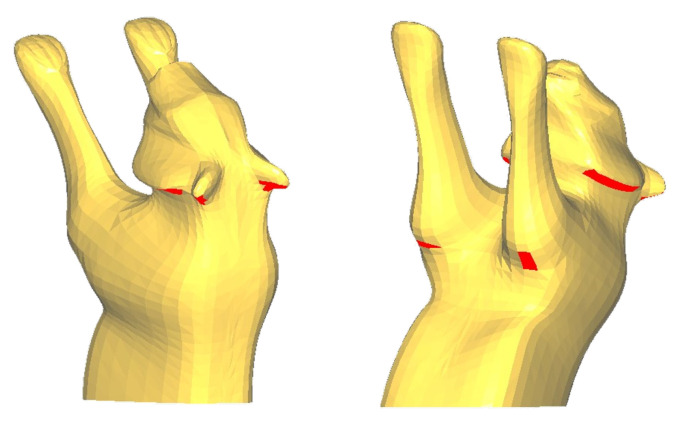
The head component of the Tiger, with the red surface area indicating the residual overhang area.

**The limitations of plane decomposition.** Our experiments reveal a fundamental limitation of planar decomposition when processing models with highly complex surfaces. While the algorithm performs well on models with relatively smooth and globally coherent geometries, it encounters an inherent bottleneck when handling models featuring localized sharp protrusions and irregular concave–convex structures, such as the intricate details on the Tiger’s head. As shown in Figure 12, the residual overhang area on the Tiger model (17.30 mm^2^; see Table 1) stems precisely from this limitation: although the clipping plane for this component is optimized to eliminate overhangs across most of its area, certain protruding local features inevitably remain as residual overhangs. The core reason is that no global cutting plane exists that can simultaneously satisfy the self-supporting requirements for all local geometries on the component. To fully resolve such issues, a transition to curved-layer decomposition strategies may be necessary.

## 5. Conclusions and Future Work

We propose a model decomposition method for multi-directional 3D printing that minimizes the overhang surface area by reducing the number of overhang patches. This method reduces the support requirements during the printing process and improves printing efficiency. The algorithm uses a heuristic approach, combining Pareto optimization and a beam search strategy to generate a series of cutting planes for model decomposition. Our algorithm demonstrates high computational efficiency and effective support optimization on various complex models. To further advance this work, the following limitations and corresponding future research directions are identified:

- Our algorithm discretizes the cutting planes. Although beam search can avoid local optima, it is essentially an improved extension of the greedy algorithm. It cannot guarantee finding the global optimum, and there may be better solutions. We note that the current objective function (Equation (9)) is constructed based on the surface patches of the initial model and cannot dynamically update the internal geometric representation during iterative decomposition, which limits the construction of an accurate continuous optimization model. Future research will consider more suitable residual model representation methods, such as converting the preceding cutting planes into patches and then dynamically updating A, B, and mask in the algorithm to establish a functional relationship between the residual overhang area and each cutting plane in the cutting sequence. Based on this, gradient-based optimization algorithms can be used to improve the optimal solution obtained by the current discrete search, thus combining the robustness of discrete sampling with the accuracy of continuous optimization.
- This algorithm does not consider the trimming range of the cutting plane. While this can prevent collisions between the print head and the printed part, it also limits the exploration of the solution space. Future research could consider the trimming range as an optimization variable. When a cutting plane cuts the model, multiple components may be generated. By performing overhang analysis on each component separately and determining the trimming range of the cutting plane based on this analysis, we can better explore the solution space. However, this may introduce collision risks. To ensure printability, an interference detection algorithm between the print head and the model (e.g., fast collision detection based on bounding volume hierarchy) needs to be introduced to automatically and safely determine the maximum effective range of each cutting plane during the optimization process, thereby fully exploring better decomposition schemes without causing collisions.
- As discussed in Section 4 (The limitations of plane decomposition), the planar decomposition method, while efficient, has inherent limitations in handling locally complex geometries. To overcome this without sacrificing print efficiency, a promising future direction is to develop a hybrid decomposition approach. This strategy would maintain planar decomposition for the model’s main body to ensure efficiency, while selectively applying curved-layer decomposition only to locally complex areas where supports are otherwise unavoidable. This approach aims to eliminate the need for supports without significantly increasing manufacturing time.

## Figures and Tables

**Table 1:** Computational statistics.

Model	Faces	Part	Computing Time (s)	Overhanging Area (mm^2^)	
				**Before**	**After**
Bunny	20,000	5	10.3	266.05	0
Kitten	10,000	5	4.6	321.09	0.71
Cup	16,462	3	3.6	114.01	0
Hand	34,198	3	5.3	798.91	0
Fox	14,282	3	5.5	58.51	0.87
Tiger	9052	5	6.2	426.81	17.30

**Table 2:** A comparison of printing time and filament length for parts manufactured by the Fixed Dir method and the Multi-Dir method.

Model	Layer Thickness (mm)	Filament Length (mm)		Print Time (min)	
		**Fixed Dir**	**Multi-Dir**	**Fixed Dir**	**Multi-Dir**
Fox	0.25	2425.30	1891.41	43	31
Bunny	0.25	6443.68	5319.68	98	78
Hand	0.25	6569.17	4996.85	112	79

## Data Availability

The original contributions presented in the study are included in the article; further inquiries can be directed to the corresponding authors.
